# Blind Detection of Broadband Signal Based on Weighted Bi-Directional Feature Pyramid Network

**DOI:** 10.3390/s23031525

**Published:** 2023-01-30

**Authors:** Shirong Guo, Jielin Yao, Pingfan Wu, Jianjie Yang, Wenhao Wu, Zhijian Lin

**Affiliations:** 1 School of Advanced Manufacturing, Fuzhou University, Fujian 362200, China; 2 College of Physics and Information Engineering, Fuzhou University, Fujian 350108, China

**Keywords:** BiFPN, signal blind detection, automatic modulation identification, short-time Fourier transform, EIOU

## Abstract

With the development of wireless technology, signals propagating in space are easy to mix, so blind detection of communication signals has become a very practical and challenging problem. In this paper, we propose a blind detection method for broadband signals based on a weighted bi-directional feature pyramid network (BiFPN). The method can quickly perform detection and automatic modulation identification (AMC) on time-domain aliased signals in broadband data. Firstly, the method performs a time-frequency analysis on the received signals and extracts the normalized time-frequency images and the corresponding labels by short-time Fourier transform (STFT). Secondly, we build a target detection model based on YOLOv5 for time-domain mixed signals in broadband data and learn the features of the time-frequency distribution image dataset of broadband signals, which achieves the purpose of training the model. The main improvements of the algorithm are as follows: (1) a weighted bi-directional feature pyramid network is used to achieve a simple and fast multi-scale feature fusion approach to improve the detection probability; (2) the Efficient-Intersection over Union (EIOU) loss function is introduced to achieve high accuracy signal detection in a low Signal-Noise Ratio (SNR) environment. Finally, the time-frequency images are detected by an improved deep network model to complete the blind detection of time-domain mixed signals. The simulation results show that the method can effectively detect the continuous and burst signals in the broadband communication signal data and identify their modulation types.

## 1. Introduction

With the rapid development of communication technologies such as 5th generation mobile networks (5G) and satellite communications, the electromagnetic spectrum, and space have become very crowded and complex due to the increasing number of different radiation sources and advanced waveforms in modern society. This has resulted in the situation that signal processing systems may be affected by multiple intentional or unintentional interfering signals injected simultaneously into their receivers [1]. Therefore, the safe use and effective control of the electromagnetic spectrum have become a priority task for radio monitoring [2]. In the cognitive radio (CR) communication system, the signal blind detection technology can achieve the accurate discovery, information restoration, and user identity adjudication of illegal authorized users, and further conduct location monitoring, spectrum suppression, and investigation and forensics to protect the safe use of radio spectrum resources by legitimate users, which is of great practical importance. In this paper, the main goal of blind detection of communication signals is the detection and preliminary automatic modulation identification (AMC) of communication signals under blind information conditions (i.e., reception of non-cooperating party signals) [3,4,5,6].

Normally, many broadband signal detection algorithms in previous studies have been based on energy detection (ED) [7]. In 1967, Ulkowitz [8] first investigated how to detect unknown deterministic signals. That is, the energy distribution is modeled as a variable non-central basis distribution random, and the threshold for determining the detection is determined by the degrees of freedom of this basis distribution and the non-central parameters. Ma et al. addressed the cognitive radio spectrum perception problem by constructing a matched filter detector using sub-user signal sequences and an energy detector using primary user signal energy and fused the results of both detectors for verdict detection, effectively mitigating the impact of sub-user out-of-connection on spectrum perception [9]. When using the spectrum-based energy detection method, simple parameters such as the number of signals, carrier frequency, and signal bandwidth can be roughly obtained. However, it is still difficult to accurately obtain the signal’s moment of appearance, modulation type, and other relevant parameters [2,10].

Deep learning (DL) techniques, developed in recent years, provide new ideas for blind detection and identification of communication signals in complex environments [11]. Some existing studies have obtained good results on AMC tasks by transforming signals into time-frequency images and then inputting them into classifiers built by neural networks [12,13]. Che et al. proposed a spatial-temporal hybrid feature extraction network for AMC, which maps wireless communication signals into spatial feature space and temporal feature space, respectively, to improve the effectiveness in the few-sample AMC task [5]. After the development in recent years, AMC techniques based on deep learning have become mature [12,14]. In 2020, the Cascaded Human-Object Interaction Recognition [15] proposed by Zhou et al. effectively improved the quality of the target detection model. In ref. [3], Cha et al. first introduced deep learning methods into a multi-signal detection task. Based on the Single Shot MultiBox Detector (SSD) target detection method [16,17], the papers performed blind detection and modulation identification on broadband signals using time-frequency images and constellation images as input of the neural network. Based on this, Dr. Rundong Li proposed an improved target detection network based on You Only Look Once version 3 (YOLOv3) to further improve the performance of deep learning methods in signal blind detection tasks and demonstrated that the You Only Look Once (YOLO), series of methods [18,19,20] outperformed Faster Region Convolutional Neural Network (FRCNN) [21,22] and traditional energy detection methods [7,10] on this task.

In summary, among the existing blind detection methods for communication signals, the speed of energy-based detection is fast, but its robustness is poor and is affected by noise and fading. On the other hand, depth-based methods are now gradually attracting the research interest of scholars and are still in their infancy, without many published research results, and there is still room for improvement in detection accuracy [10]. Moreover, the existing deep learning-based signal blind detection methods do not achieve good results for testing under low Signal-Noise Ratio (SNR) conditions. This is mainly because, with the addition of complex fading channel conditions and non-smooth undulating noise environments, the signals received at the receiver side are subject to complex and diverse interference. Furthermore, the direct application of existing deep neural network models to signal sequences or their feature maps does not guarantee their robustness. Based on the deep learning methods that have been proven to perform best for the signal blind detection task, this paper will improve the deep learning methods used for signal blind detection in terms of comprehensive performance and especially low signal-to-noise ratio.

In this paper, we discuss the problem of blind detection and modulation identification on broadband communication signals based on deep learning. Based on previous research, this paper proposes a blind detection method for wideband signals based on BiFPN and Efficient-Intersection over Union (EIOU). The main contributions of our work are as follows.

(1) A system model for transmitting time-domain mixed signals in a non-cooperative reception environment is developed, and the signals are analyzed at the receiver side by short-time Fourier transform (STFT) for time-frequency analysis to extract the normalized time-frequency images and the labels of the relative positions of each modulated signal in the image.

(2) A target detection model for time-domain mixed signals is built based on YOLOv5 to learn the features of the signal time-frequency distribution image dataset to train the model. To the best of our knowledge, this paper introduces the YOLOv5 method improved by BiFPN and EIOU to the field of signal blind detection for the first time.

(3) Based on the dataset generated in this paper, we validate the proposed method through extensive experiments. The results show that the improved method outperforms the existing methods in terms of comprehensive performance, especially under low SNR conditions.

The rest of the paper is organized as follows. Section 2 introduces the signal model, the dataset, and its automatic labeling method. Section 3 presents the structure of the relevant algorithmic model and the description of the model improvement in this paper. Section 4 presents the dataset configuration, performance metrics, and comparative results of our experiments. Finally, the conclusion and future work are given in Section 5.

## 2. Methods

**Figure 1:**
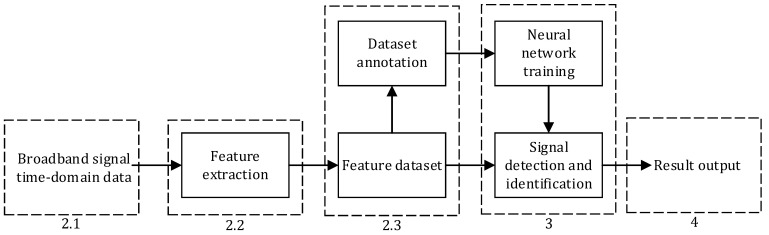
Deep learning method workflow block diagram.

In this paper, blind detection of broadband communication signals is formulated as a deep learning-based target detection task, aiming to obtain the moment of occurrence, modulation type, and other relevant parameters of each constituent signal in the composite signal. The workflow is shown in Figure 1. Next, in this paper, our signal model and algorithm flow are presented in parts according to the indications of the dashed boxes in the flow block diagram.

**Figure 2:**
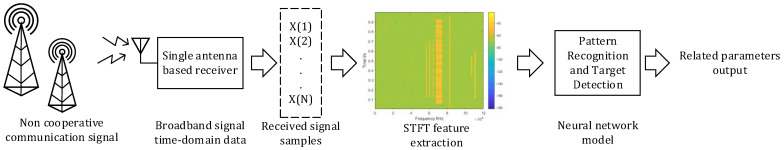
The paper deep learning signal detection framework.

The signal detection framework is shown in Figure 2, where a single receiver receives communication signals from multiple non-cooperative transmitters. Next, the received signal sequences are integrated into a matrix with a time-domain superposition between individual signal sequences. Then, STFT is applied to the integrated signal matrix to extract its time-frequency features, and a series of images that can be judged by vision is obtained through normalization. After that, the series of images are made into data sets and input into the target detection algorithm (DL method) proposed in this paper for training. Finally, a model that can detect relevant parameters can be obtained.

### 2.1. System Model

According to the first half of the frame shown in Figure 2 (the first three steps), we perform signal modeling. The communication process of a general wireless communication system can be represented as

(1) $$r_{0}(t)=s(t)h(t)+n(t)$$

where *r_0_*(*t*) is the signal received at the receiver; *s*(*t*) is the modulated signal transmitted at the transmitter; *h*(*t*) is the channel response, and *n*(*t*) is the additive Gaussian white noise at the receiver and in the channel with a mean of 0 and a variance of *δ*^2^. We assume that the receiver detects transmitter signals consisting of n modulation types and treats these signals as time-domain mixed signals. At this point, the communication process is expressed as

(2) $$r(t)=\sum_{i=1}^{k}s_{i}(t)h_{i}(t)+n(t)(k=1,2,\ldots ,N_{s})$$

where *r*(*t*) denotes the mixed signal detected and received by the receiver in the time domain detection process, *s_i_h_i_*(*t*) denotes the *i*th target signal, the number of modulation types of radiation source signals that can be received by the receiver during operation is denoted by *k*, and the number of all possible signal modulation types in the target source is denoted by *N_s_*. We assume that each target signal contains only one modulation parameter. 

### 2.2. Feature Extraction

STFT essentially uses a sliding window symmetric about the center to observe the signal inside the window and obtains the spectrum of that time slot by performing Fourier transform on the signal inside the window. Finally, the time-frequency image of the target signal is obtained by stitching. The operation steps on the received signal *r*(*t*) are as follows: First, the window function is moved to the initial position of the signal. The center of the window function is *τ*_0_, and the signal in the window is represented as

(3) $$y(t)=r(t)w(t-\tau_{0})$$

where *w*(*t*) is the window function and *r*(*t*) is the source signal *y*(*t*). Then the Fourier transform is performed, and the spectral distribution of the first segmented sequence *R*(*w*) is obtained as

(4) $$R(w)=F(y(t))=\int_{-\infty }^{+\infty }r(t)w(t-\tau_{0})e^{-jwt}dt$$

The window function is moved to *τ* after the first Fast Fourier Transform (FFT) operation, where the distance it is moved is called the bounce size. During the shift, a certain overlap between the two windows should be ensured, i.e., the width of the window should be larger than the distance moved, which is called “overlap”. The STFT of the signal is obtained by continuously sliding the window and repeating the above operation.

(5) $$S\mathrm{TFT}(w,\tau )=F(r(t)w(t-\tau ))=\int_{-\infty }^{+\infty }r(t)w(t-\tau )e^{-jwt}dt$$

In this paper, according to our experience. We set the window length to 1024. The window type is Hamming window. Each step length is 1. 

In addition, in order to avoid the bias of feature quantities because of the difference in input signal power, the magnitude of the short-time Fourier time-frequency image is normalized.

**Figure 3:**
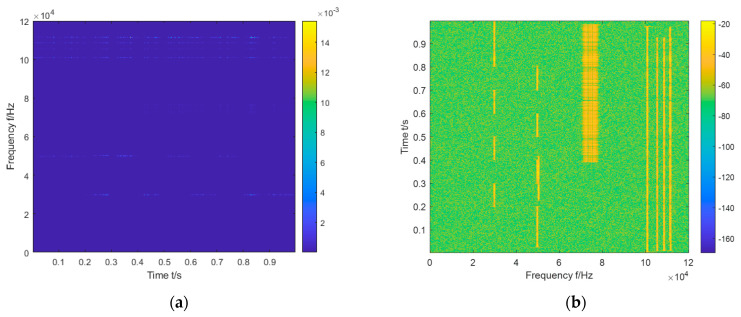
Image (**a**) is the original time-frequency image, and image (**b**) is the normalized image.

As shown in Figure 3, the horizontal coordinate of the image on (a) is time, and the vertical coordinate is frequency; the horizontal coordinate of the image on (b) is the frequency, and the vertical coordinate is time. According to the different RGB values of each pixel, it can reflect the energy contained in the corresponding time and frequency of this pixel. Compared with previous images, the normalized image can make energy easier to locate and identify its features through vision. The time-frequency distribution image is resized to 875 × 656 × 3 and then passed to the subsequent model for target detection (875 × 656 denotes the length × width format of the image, and 3 denotes the number of RGB channels).

### 2.3. Dataset Generation and Automatic Annotation

By the above method, we extract the individual signals at the receiving end for time-domain superposition, record the time and frequency parameters of each signal at the time of superposition, synthesize the composite signal with time-domain superposition, and then perform STFT transformation to obtain its two-dimensional time-frequency image. Based on the previously recorded time, center moment, and frequency parameters, we map them to the two-dimensional time-frequency image to obtain the coordinate information of the real prediction frame of each signal in the graph and then perform automatic labeling of the data.

**Figure 4:**
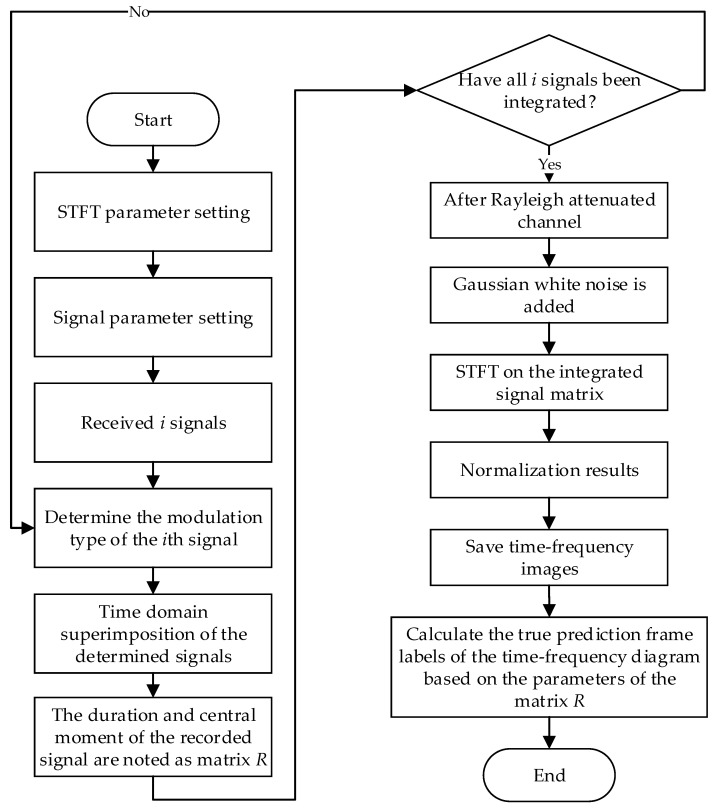
Block diagram of dataset generation and automatic annotation procedure.

The block diagram of the procedure for dataset generation and automatic labeling is shown in Figure 4. It is worth mentioning that we simulate Rayleigh fading channels utilizing Zheng’s model [23]. Regarding the simulation of this model, all experiments in this paper use parameters with a superimposed sine wave number of 4 and a maximum Doppler shift of 100 Hz. By this method, we can simplify the dataset acquisition procedure and improve the efficiency of dataset acquisition without reducing the data validity.

## 3. Deep Learning-Based Target Detection

Currently, deep learning is the most common and efficient means to solve the target detection problem. The existing target detection methods are divided into two main categories: two-stage and single-stage [24]. The two-stage method is a deep convolutional network based on candidate regions. Possible candidate blocks containing the detection target are first generated, and then the candidate blocks are classified and corrected to obtain detection frames for target detection. The common algorithms are RCNN (Region CNN) [25] and others. These methods have a high detection accuracy but a low detection speed. Single-stage methods are target detection based on deep convolutional networks with regression computation using end-to-end target detection methods such as YOLO series [18,19,20] and so on. These methods have faster detection speeds and can meet real-time requirements. Considering the detection efficiency, we prefer the single-stage model to complete the signal-blind detection task. With the progress of recent research advances, the single-stage YOLO series methods can even achieve higher performance than the two-stage RCNNs for some tasks [10]. Therefore, the YOLO series of target detection models are very suitable for us.

In this part, based on the standard YOLOv5 network structure, we propose a method to improve the target detection model using a weighted BiFPN in order to achieve simple and fast multi-scale feature fusion and introduce the EIOU loss function to achieve higher accuracy signal detection.

### 3.1. Basic YOLOv5 Network Overview

**Figure 5:**
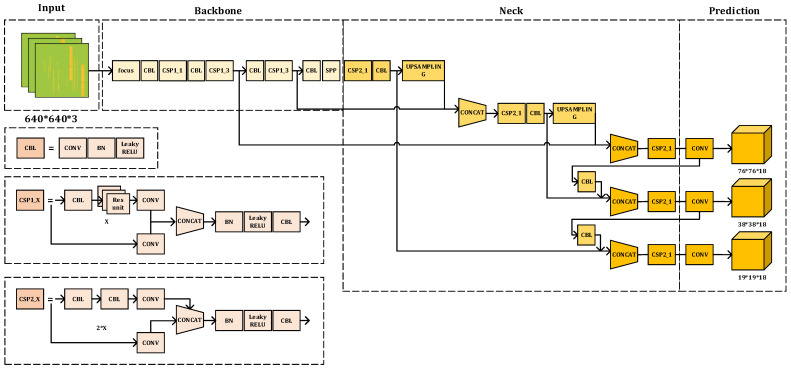
YOLOv5 network structure diagram.

YOLOv5 is a single-stage target detection network proposed by Ultralytics LLC. As the most mature and stable target detection network in the YOLO series at present, it is the product of improvements based on YOLOv4 and YOLOv3 [18,19,20]. After learning the advantages of the previous versions and other networks, YOLOv5 changes the previous YOLO target detection algorithm’s characteristics, i.e., faster detection but not high accuracy. This network improves detection accuracy and real-time performance, which not only meets the need for real-time image detection but also has a smaller structure. Therefore, in this paper, we use the standard YOLOv5 as the target detection model, and the network structure is shown in Figure 5 [20], whose network model is divided into four parts: input, backbone, neck, and prediction head. 

Firstly, the collated dataset images are input to the network after preprocessing operations such as data enhancement and scaling. Then, the focus module downsampling, CSP structure, and SPP pooling layer are used to extract the image features and compose the main part. The focus module can segment the input image into four parts to piece together a 12-dimensional image with features and obtain 3 × 3 feature information from it to generate a 32-dimensional image that contains feature information. The focus module downsampling has the advantage of preserving image information and reducing computation, and its use can improve the overall training speed. The structure of CBL consists of a convolution (Conv) layer and a batch normalization (BN) layer, while the signal is passed to the activation function layer. The Conv module in SPP has the advantage of achieving a reduced number of input feature maps. By using the Conv module, the maximum set of subsamples can be covered in the case where there are three different convolution kernels, and the results are stitched to the input feature maps by channel. YOLOv5 has the advantage of implementing SPP by using convolution kernels of the same step size. Therefore, although the feature maps obtained by this network are of the same size, the regional sensitivities vary, and the features are also fused. Thirdly, the neck part uses the structure of a Path Aggregation Network (PANet) combined with Feature Pyramid Networks (FPN) as the feature aggregation layer of the whole network architecture, and the function of this part is to obtain the multi-scale information of the image. Finally, we use three loss functions to form the prediction head part as a way to calculate feature classification, feature localization, and confidence loss and to make accurate network predictions by non-maximal rejection (NMS). The output of the algorithm consists of a vector of prediction frame categories, confidence levels, and coordinate positions. This network structure is used as a basis for improving the characteristics of the signal blind detection task in this paper.

### 3.2. Improved Neck Network

The weighted BiFPN is an efficient neck network structure proposed by Tan et al. in EffificientDet, a single-stage target detection model [26]. The network, which also belongs to convolutional neural networks, introduces learnable weights to learn the importance of different scale features extracted from an efficient backbone network and iteratively uses top-down and bottom-up sampling methods to fuse multi-scale features. Then, the fused features are fed into the class prediction network and box prediction network for detection. Finally, we note that the performance of the network improves with increasing network depth based on the superposition of BiFPN. The structure is as follows. 

**Figure 6:**
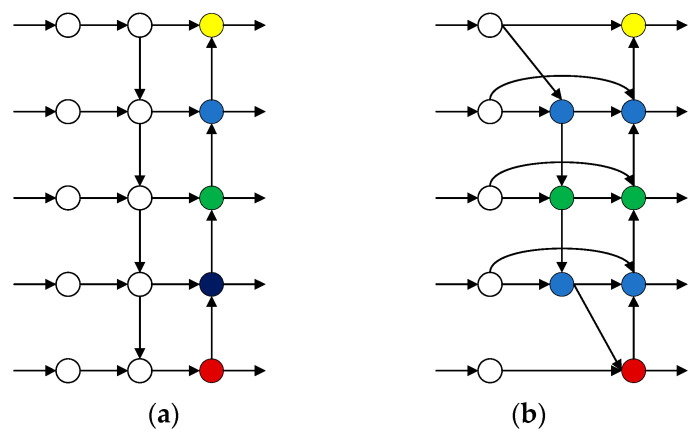
PANet (**a**) and BiFPN (**b**) base module structure.

As shown in Figure 6, compared with the PANet structure, the BiFPN base block structure contains not only horizontal connections from left to right and vertical connections from top to bottom and bottom to top, but also there are cross-scale connections. In this way, we can extract more features at different levels for fusion so that we can have attention to the whole situation and will not easily miss any information. Moreover, BiFPN blocks can be stacked as many times as needed. BiFPN blocks can be represented as

(6) $$P_{i}P_{i+1}\ldots P_{i+n}=f(C_{i}C_{i+1}\ldots C_{i+n})$$

where *n* is the number of features used for feature fusion, *C* is the input features of a layer, *P* is the output features of a layer, and *f* is a function of the feature fusion process.

**Figure 7:**
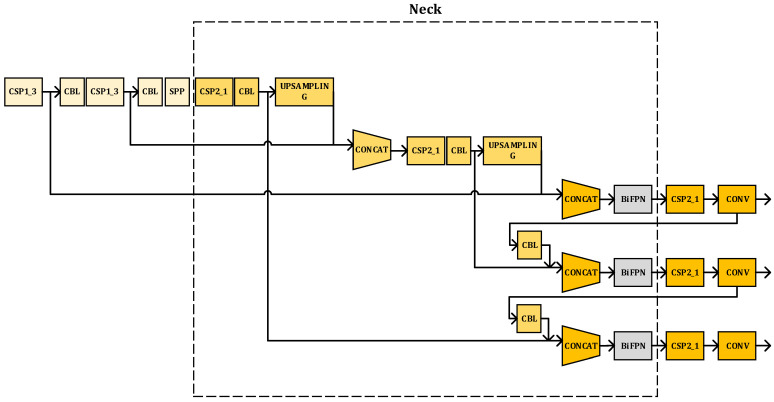
Schematic diagram of the improved neck network structure.

Based on the above, we change the neck network of standard YOLOv5 from the original PANet to a weighted BiFPN. The improvement of the network structure is shown in Figure 7. The neck network structure of YOLOv5 is mainly responsible for feature extraction and fusion and introduces learnable weights by accessing the BiFPN module to learn from the efficient backbone features extracted from the network at different scales. Then, repeatedly employing top-down and bottom-up sampling methods to fuse multi-scale features, thus enhancing the fusion of features. That is, introducing BiFPN can help us focus on the big picture and not miss any information. In the time-frequency images generated by the system modeling in this paper, most of the signals to be detected are rectangular and strip-shaped. Due to the aliasing of continuous signals and burst signals in the time domain, each target signal to be detected in the figure globally is more relevant, so we think this improvement is helpful for our task.

### 3.3. Improved Loss Function

The standard Intersection over Union (IOU) loss function is shown in Equation (7) below when the prediction frame A does not intersect with the real frame B, i.e., A ∩ B = Ø when the loss function has no gradient. In addition, the IOU may be different when the prediction frame and the real frame have the same size, and the IOU loss function cannot distinguish between these two cases.

(7) $$\mathrm{IOU}\text{\_}\mathrm{Loss}=1-\mathrm{IOU}=1-\frac{A\cap B}{A\cup B}$$

In order to overcome the above problems, new IOU loss functions have been continuously developed by researchers, and their development has resulted in Generalized-IOU (GIOU), Distance-IOU (DIOU), Complete-IOU (CIOU) and Efficient-IOU (EIOU), which are loss functions that can effectively improve the target detection performance [27,28,29,30]. Among them, EIOU is the current relatively advantageous one, which is developed based on DIOU and CIOU. The formulas of DIOU and CIOU loss functions are as follows.

(8) $$\mathrm{DIOU}\text{\_}\mathrm{Loss}=1-(\mathrm{IOU}-\frac{\rho^{2}(b,b^{gt})}{c^{2}})$$

(9) $$\mathrm{CIOU}\text{\_}\mathrm{Loss}=1-(\mathrm{IOU}-\frac{\rho^{2}(b,b^{gt})}{c^{2}}-\alpha \gamma )$$

where, $\alpha =\frac{\gamma }{1-\mathrm{IOU}+\gamma }$, $\gamma =\frac{4}{\pi^{2}}{\left(\arctan\frac{w^{gt}}{h^{gt}}-\arctan\frac{w}{h}\right)}^{2}$, *b* and *b^gt^* respectively are the middle points of the prediction box and the real box; *w* and *w^gt^* respectively are the width of the prediction box and the real box; *h* and *h^gt^* respectively are the height of the prediction box and the real box; *ρ* is the Euclidean distance between the two middle points; *c* is the diagonal distance of the best that can include the prediction and real box.

As shown in Equation (10) below, EIOU takes into account not only the centroid distance and aspect ratio but also the real differences in width and height of the target and anchor boxes. The EIOU loss function directly minimizes these differences and accelerates the convergence of the model.

(10) $$\mathrm{EIOU}\text{\_}\mathrm{Loss}=1-(\mathrm{IOU}-\frac{\rho^{2}(b,b^{gt})}{c^{2}}-\frac{\rho^{2}(w,w^{gt})}{c_{w}^{2}}-\frac{\rho^{2}(h,h^{gt})}{c_{h}^{2}})$$

When the SNR is too low, the overall texture of the time-frequency image becomes blurred, and the detection frame obtained by the target detection model for the signals with the time-domain aliasing relationship with each other is more prone to false detection or omission of detection. In Section 3.2, we describe the method to enhance the neck network by replacing the PANet module with a BiFPN module. The introduction of the BiFPN module allows the original model to discover more features, thereby adding more candidate sample frames. However, this improvement increases the number of correctly detected sample frames but also increases the number of false detections or missed detections. Compared with the previous CIOU loss function, the EIOU loss function takes into account the overlapping area, the distance between the center points, and the real difference in length, width, and side length. Based on CIOU, the fuzzy definition of aspect ratio and the problem of sample imbalance in bounding box regression are solved. Therefore, It is believed that the introduction of the EIOU loss function can inhibit the increase in false detection frames caused by the introduction of the BiFPN module to a certain extent.

### 3.4. Transfer Learning

**Figure 8:**
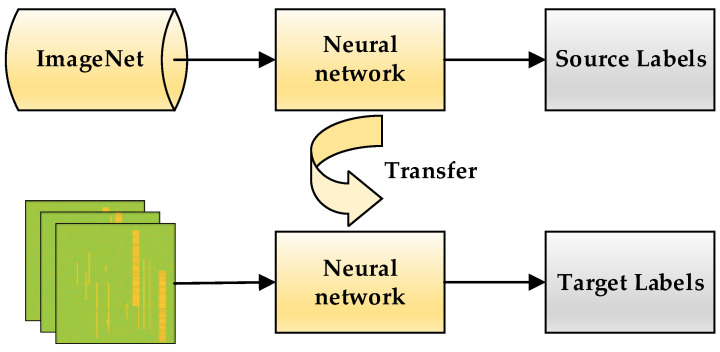
Schematic diagram of transfer learning method.

For all the neural network models used in this paper, we use the transfer learning approach. The neural network models used are pre-trained on the ImageNet dataset [31], and the last fully connected layer of the network is changed from 1000 to 4 layers. The network is initialized using pre-trained parameters and trained by fine-tuning the entire network parameters with new data generated by the method in Section 2 to improve the performance of the network on the desired task. All other networks are frozen, and all network parameters are fine-tuned according to the pre-trained model parameters. The transfer learning approach based on fine-tuning each layer of the network is shown in Figure 8. A transfer learning approach based on fine-tuning entire layers of the network to train a new dataset with weights that have been trained on ImageNet.

## 4. Experiment

In this section, we first determine the parameters and experimental conditions of the data set required for the experiment, secondly determine the experimental evaluation metrics, and finally conduct model comparison experiments and analyze the results.

### 4.1. Data set Configuration and Experimental Conditions

#### 4.1.1. Training Dataset Parameters

Based on the signal modeling, data generation, and automatic labeling methods mentioned in Section 2, the communication model is built by Matlab 2020b, and the parameters of the two-dimensional time-frequency image data set for training are shown in Table 1.

In the final training data set composition, we generate 20,000 time-frequency images, of which 80% are divided into the training set and 20% into the validation set.

#### 4.1.2. Test Data Parameters

**Figure 9:**
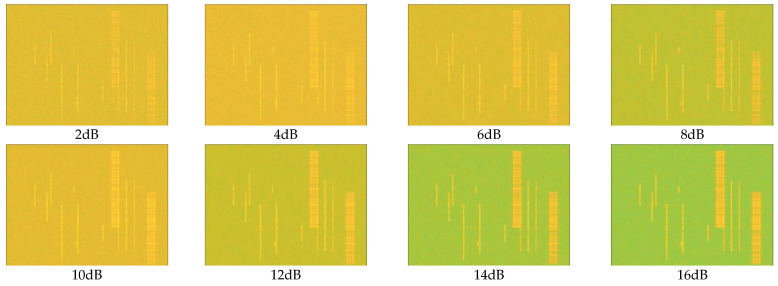
Comparison of the differences in the time-frequency diagrams generated at each SNR.

The signal parameters of the independent test data are the same as those of the training dataset but are not involved in training at all. The different parameters are mainly the target SNR range of the test set. In the test set, SNR varies from 0 to 16 dB in 2dB steps. From Figure 9. we can visualize the effect of SNR on the generated time-frequency diagram. With the reduction in SNR, noise has more and more influence on the definition of a time-frequency image, and it is more difficult to analyze the signal in the image through vision. The number of time-frequency images in the test set is 1000 for each SNR condition.

#### 4.1.3. Inference Parameters at Training

For training, the time-frequency image is first scaled into a 640 × 640 image input to the network, the maximum number of training cycles (epochs) is 100, the batch size of each update is 16, and the SGD (Stochastic Gradient Descent) optimization algorithm [32] with default parameters is used for training. In the experiments, the algorithm is trained and tested using NVIDIA Titan V 12G, and the network model is implemented on PyTorch1.11.0 framework using Python language. Other important environment configurations include opencv4, CUDA 11.6, and Python 3.9.12.

### 4.2. Model Evaluation Indicators

In the analysis of the comparison of experimental results of signal detection, the following three performance indicators [2] are mainly used in this paper.

1. Detection probability *P_d_*: *P_d_* is the ratio of the number of detected real signals to the total number of actual signals. Let the total number of actual signals to be detected by *N* and the number of detected target signals be detected by *M,* which can be expressed as *M*=*M_T_*+*M_F_*, where the number of real signals is *M_T_*, and the number of false results with no signal is *M*, then *P_d_* = *M_F_* / *N*.

2. False alarm probability *P_f_*: *P_f_* is the ratio of the number of detected spurious signals to the total number of detected signals, that is, *P_f_* = *M_F_* / *M.*

3. The average error of signal parameters *E_avg_*: *E_avg_* is to count the frequency, bandwidth, and burst start and end time parameters for all detected real signal targets: $f_{c0}^{i}$, $B_{w0}^{i}$, $t_{s0}^{i}$, and $t_{e0}^{i}$ with the corresponding real parameters: $f_{c}^{i}$, $B_{w}^{i}$, $t_{s}^{i}$, and $t_{e}^{i}$, then the average relative error between them is calculated by the formula (11).

(11) $$E_{avg}=\frac{1}{4M_{T}}\sum_{i=1}^{M_{T}}\left(\frac{\left|f_{c}^{i}-f_{c0}^{i}\right|}{B_{w}^{i}}-\frac{\left|B_{w}^{i}-B_{w0}^{i}\right|}{B_{w}^{i}}-\frac{\left|t_{c}^{i}-t_{s0}^{i}\right|}{T_{}^{i}}+\frac{\left|t_{e}^{i}-t_{e0}^{i}\right|}{T_{}^{i}}\right)$$

where *T^i^* is the true signal duration, and $B_{w}^{i}$ is the true signal bandwidth.

In experiments where the performance gap is obvious, we will directly use mean Average Precision (mAP) [33] as the evaluation index, as shown in the following formula:

(12) $$P(k)=\frac{TP}{TP+FP}\times 100\%$$

(13) $$R(k)=\frac{TP}{TP+FN}\times 100\%$$

(14) $$mAP=\frac{1}{C_{s}}\sum_{M=i}^{N}P(k)\Delta R(k)$$

where *TP* is the number of correctly identified signal samples, *FP* is the number of incorrectly or unidentified signal samples, and *FN* is the number of incorrectly identified signal sample targets. *C_s_* is the number of categories of signal samples; *M* and *N* represent the number of IOU thresholds and IOU thresholds; *P*(*k*) and *R*(*k*) is the precision and recall rates.

### 4.3. Experiments and Results Analysis

The experiments were started according to the above configuration, and all networks in the experimental process were subjected to transfer learning in combination with pre-trained weight models according to the method introduced in Section 3.4. After testing, this method can improve the training effect without increasing the training cost in the task of this paper.

**Figure 10:**
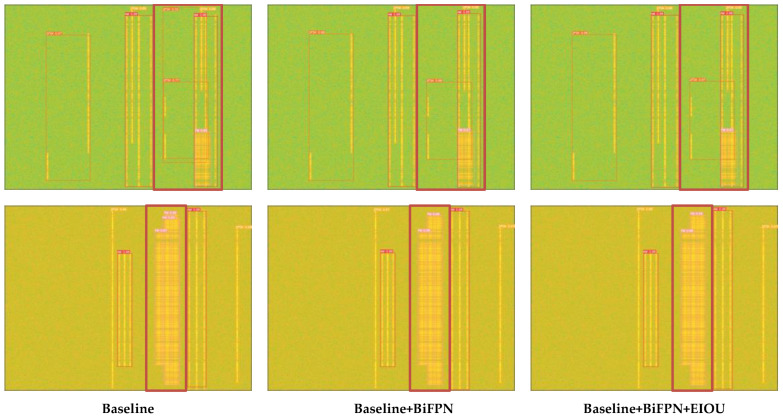
Comparison of the detection of different models.

In the experiments, we tested the performance metrics under each SNR according to the above dataset configuration and created a line graph with SNR as the horizontal coordinate and specific performance metrics as the vertical coordinate for analysis. Figure 10 shows the output of different models for the same graph during the experiment, and it can be easily seen that the improved model avoids some of the initial false detection cases. More specifically, as indicated by the thick red rectangular box in the figure, the locations where the test results change at different stages are shown here. In the detection result of the Baseline model, there are three target boxes in the thick red box of the first image, and one of the target boxes clearly overlaps the other target boxes. We can easily determine that the extra overlapping target box is caused by wrong detection. The improved detection result leaves only two correct target boxes, which proves that our improvement is effective. In addition, we also calculated the average value of each performance index between SNR 0 and 16 dB, as shown in Table 2.

The average performance of the four methods shows that the performance of the proposed method in this paper is improved in all aspects, which proves the effectiveness of the method.

**Figure 11:**
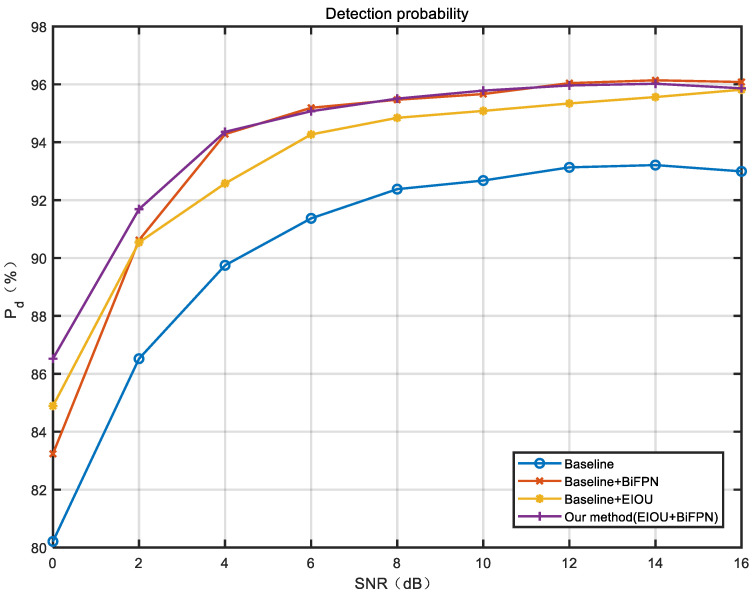
Experimental results of detection probability under different signal-to-noise ratios.

Figure 11 shows the comparison of the detection probabilities obtained for the models at different stages of this paper with different signal-to-noise ratios of the test set. As shown in Figure 11, we compare the performance of the four neural network models under different SNR conditions. Since the model with the best results in previous studies is the YOLOv3 + CIOU model proposed in the article [10], and through our test, YOLOv5 performs better than YOLOv3 in this task. Therefore, we use the YOLOv5 + CIOU model as our Baseline. Overall, the detection probability increases with the increase in signal-to-noise ratio (SNR). The experimental results demonstrate that the improved model with the added BiFPN and EIOU module can effectively improve the performance of target detection, and the detection probability increases by about 3–4% in the range of SNR of 0-16 dB. The model improved by the EIOU loss function is also able to improve the target detection performance, with a detection probability improvement of about 2–3% in the range of 0–16 dB. Obviously, BiFPN is more efficient in terms of improvement of detection probability compared to EIOU. Finally, by comparing the final model proposed in this paper with the model improved by the Baseline + BiFPN module, we find that in the range of SNR of 4–16 dB, there is no marked difference in the performance between the two models in terms of detection probability, and the effects are considered to be same. However, in the range of SNR of 0–4 dB, the final model proposed in this paper has significantly higher performance with an increase in detection probability of about 1–3%. Therefore, we can conclude that the final model proposed in this paper has an overall improved performance in detection probability compared to the baseline model and Baseline + EIOU model and has higher robustness and improved performance in detection probability at low SNR compared to the Baseline + BiFPN model.

**Figure 12:**
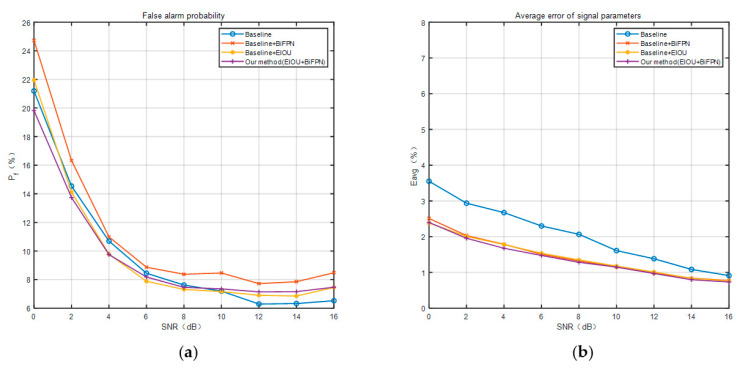
Experimental results of false alarm probability (**a**) and average error of signal parameters (**b**) at different signal-to-noise ratios.

Figure 12 shows the comparison of the false alarm probability (a) and the average error of the signal parameters (b) obtained for the models at different stages of this paper with different signal-to-noise ratios of the test set. Similar to Figure 11, we also compare the performance of the four neural network models at different stages. First, we can find that the false alarm probability and the average error of signal parameters decrease as the SNR increases. Then, in the comparison of the false alarm probability, the Baseline + BiFPN module has the highest, which proves to be the worst performance in this regard. This shows that although the detection probability is improved by adding the BiFPN module, the false alarm probability also increases. Moreover, the Baseline + EIOU model clearly causes a lower false alarm probability than the Baseline + BiFPN model. The addition of EIOU effectively reduces the false alarm probability in the low SNR case, as well demonstrated in Figure 12. Therefore, the suppression of false alarm probability by EIOU is evident, and we proceeded to analyze the results of the final BiFPN + EIOU improved model on this basis. On average, the final method proposed in this paper has the lowest one, which proves to be the best performance in this regard. In other words, the final method can improve the increased false alarm probability with the addition of the BiFPN module and has excellent performance. Finally, in the comparison of the average error of signal parameters, we find that the final method proposed in this paper is the lowest and the baseline method is the highest. The BiFPN improved model and the EIOU improved model are in the middle of this performance, and the data for both are very similar, leading to almost overlapping curves. Compared with the baseline method, the final method proposed in this paper shows the greatest improvement at 0 dB, with a reduction in about 1%. In the range of SNR of 0-16dB, the average error of signal parameters is reduced by about 1–0.25%, which can prove its excellent performance.

Finally, we conducted comparison experiments among the proposed method with the FRCNN method to demonstrate that our model still has advantages in the face of two-stage target detection models. The results are shown in Table 3. It is worth mentioning that due to the large arithmetic power required by FRCNN, we generated an additional training set consisting of 2000 time-frequency images, and the other parameters of the dataset are kept consistent. In the experiments, the FRCNN and our method used the same training dataset. With the assurance of model convergence, we used mAP to measure the effectiveness of the model. Because of the huge disparity in mAP, we did not evaluate these two models with other metrics anymore. In addition, we also tested the mAP of the SSD model [18] and compared its results with the results of the model proposed in this paper.

Obviously, by comparing the performance of mAP with SSD and FRCNN, we conclude that our model works much better than FRCNN and SSD.

## 5. Conclusions

According to the application scenarios of signal blind detection, we first propose a high-performance target detection model combined with STFT time-frequency image feature extraction to test the effect of different SNRs on target detection in a Rayleigh fading channel + Non-smooth undulating noise environment. Subsequently, we improved the YOLOv5 model, which combines BiFPN and EIOU to accomplish the target detection task for signal blind detection of STFT time-frequency images. According to the requirements of practical applications, we detect four modulation types, AM, FM, MFSK, and MPSK, and then compare the performance of these four types of detection with that of the baseline model detection to check the differences between the improved model and the original model. Finally, through comparative experimental studies, we demonstrate the superiority of our model. Therefore, we strongly believe that this important area will be a fruitful research direction, but we have just touched the tip of the iceberg. We have provided a novel strategy to process signals and hope to attract some attention from the relevant area.

In our future work, we will consider blind detection on broadband communication signals in more complex environments, such as Multiple Input Multiple Output (MIMO) communication systems [34,35]. We will consider model compression and acceleration using the Filter Pruning via Geometric Median (FPGM) algorithm and TensorRT framework.

## Figures and Tables

**Table 1:** Training dataset parameter setting.

Parameter Name	Parameter Setting
Broadband data sampling rate	240 kSps
Time-frequency images horizontal coordinate range (bandwidth range)	0–120 kHz
Time-frequency images vertical coordinate range (time duration)	0–1 s
Time-frequency images size	875 × 656 × 3
Target signal duration	0.2–1 s
Number of target signals per image	2–8
Target signal modulation mode	AM FM MFSK MPSK
Signal-to-noise ratio range	0–16dB
channel simulation conditions	Rayleigh fading channel + Non-smooth undulating noise

**Table 2:** Comparison of the average parameter indicators of the four methods.

Methods	Average SNR Performance between 0 and 16 dB		
	P_d_	P_f_	E_avg_
Baseline	90.25%	9.87%	2.06%
Baseline + BiFPN	93.63%	11.31%	1.43%
Baseline + EIOU	93.21%	9.94%	1.42%
**Baseline + BiFPN + EIOU**	**94.09%**	**9.79%**	**1.38%**

**Table 3:** Comparison of the mAP of YOLOv5 and other methods.

Methods	YOLOv5 + BiFPN + EIOU	SSD	FRCNN
mAP	92.46%	84.11%	77.10%

## Data Availability

Not Applicable.
